# The Behavioral Consequence of Phenylketonuria in Mice Depends on the Genetic Background

**DOI:** 10.3389/fnbeh.2016.00233

**Published:** 2016-12-20

**Authors:** Vibeke M. Bruinenberg, Els van der Goot, Danique van Vliet, Martijn J. de Groot, Priscila N. Mazzola, M. Rebecca Heiner-Fokkema, Martijn van Faassen, Francjan J. van Spronsen, Eddy A. van der Zee

**Affiliations:** ^1^Molecular Neurobiology, Groningen Institute for Evolutionary Life Sciences, University of Groningen Groningen, Netherlands; ^2^Department of Pediatrics, Beatrix Children's Hospital, University Medical Center Groningen Groningen, Netherlands; ^3^Laboratory Medicine, University of Groningen, University Medical Center Groningen, Netherlands

**Keywords:** strain, pah mutation, novel object recognition, spatial memory, phenylketonuria, mouse models, genotype, phenotype

## Abstract

To unravel the role of gene mutations in the healthy and the diseased state, countless studies have tried to link genotype with phenotype. However, over the years, it became clear that the strain of mice can influence these results. Nevertheless, identical gene mutations in different strains are often still considered equals. An example of this, is the research done in phenylketonuria (PKU), an inheritable metabolic disorder. In this field, a PKU mouse model (either on a BTBR or C57Bl/6 background) is often used to examine underlying mechanisms of the disease and/or new treatment strategies. Both strains have a point mutation in the gene coding for the enzyme phenylalanine hydroxylase which causes toxic concentrations of the amino acid phenylalanine in blood and brain, as found in PKU patients. Although the mutation is identical and therefore assumed to equally affect physiology and behavior in both strains, no studies directly compared the two genetic backgrounds to test this assumption. Therefore, this study compared the BTBR and C57Bl/6 wild-type and PKU mice on PKU-relevant amino acid- and neurotransmitter-levels and at a behavioral level. The behavioral paradigms were selected from previous literature on the PKU mouse model and address four domains, namely (1) activity levels, (2) motor performance, (3) anxiety and/or depression-like behavior, and (4) learning and memory. The results of this study showed comparable biochemical changes in phenylalanine and neurotransmitter concentrations. In contrast, clear differences in behavioral outcome between the strains in all four above-mentioned domains were found, most notably in the learning and memory domain. The outcome in this domain seem to be primarily due to factors inherent to the genetic background of the mouse and much less by differences in PKU-specific biochemical parameters in blood and brain. The difference in behavioral outcome between PKU of both strains emphasizes that the consequence of the PAH mutation is influenced by other factors than Phe levels alone. Therefore, future research should consider these differences when choosing one of the genetic strains to investigate the pathophysiological mechanism underlying PKU-related behavior, especially when combined with new treatment strategies.

## Introduction

Transgenic and knockout/ knock-in mice are used to investigate the consequence of genetic mutations to understand the human biological system, especially in a diseased condition. It is progressively acknowledged that the strain of these mice highly influences the outcome of the gene mutation(Holmes et al., 2002; Doetschman, 2009; Sittig et al., 2016; Alam et al., 2017). Nevertheless, identical gene mutations in different strains are often still considered equals in various disciplines. A striking example is the mouse model used in the field of phenylketonuria (PKU, OMIM 261600). PKU is an inheritable metabolic disorder characterized by high concentrations of the amino acid phenylalanine (Phe) in blood and brain caused by mutations in the gene that encodes for the enzyme Phe hydroxylase (PAH, EC 1.14.16.1). This mutation results in a loss of catalytic activity of the enzyme and, as a consequence, the conversion of Phe to tyrosine is disrupted. In untreated patients, these raised concentrations of Phe are associated with symptoms such as a severe intellectual disability, disruptions in motor performance, mood swings, anxiety, depression disorders, and epilepsy (Blau et al., 2010). The mouse model of PKU mimics the PKU patients through a chemically induced point mutation in the gene encoding for the enzyme PAH. Originally, this point mutation was described for the black and tan, brachyury (BTBR) mouse (Shedlovsky et al., 1993). However, the wild-type (WT) mice of the BTBR strain were found to have difficulties with breeding and displayed abnormalities in brain morphology and behavior, thus limiting their suitability for preclinical research (Wahlsten et al., 2003; Ding et al., 2006; MacPherson et al., 2008; Jones-Davis et al., 2013). Therefore, The BTBR PKU mouse was crossed back on a C57Bl/6JRj (referred to as B6 hereafter) background. As a result, both strains are currently used in PKU studies, often without justification. Without fully understanding the influence of the genetic background on behavior and physiology, notably behavioral results in these PKU studies can be difficult to interpret.

Various studies highlight the phenotypical difference in behavior between BTBR and B6 WT, as the BTBR is often used in autism research. For example, novelty induced activity is greater in BTBR WT than B6 WT (Cabib et al., 2003; Moy et al., 2007; Molenhuis et al., 2014) but decreased in home-cage conditions (Molenhuis et al., 2014). Furthermore, motor performance of the BTBR WT is inferior to the B6 WT on the rotarod (Nadler et al., 2006; Moy et al., 2007). However, mixed results are described for the differences between the strains in anxiety-related behavior and learning and memory. For anxiety-related behavior, no differences (Cabib et al., 2003), a reduction (Molenhuis et al., 2014), and an increase of anxiety-related behavior of BTBR WT (Moy et al., 2007) compared to B6 WT are reported. Similar contradicting results are described for learning and memory. Some articles show an intact ability to master a short-term or long-term memory task by both backgrounds (Cabib et al., 2003; Molenhuis et al., 2014). Others report memory deficits in for instance short-term novel object memory in the B6 WT (Cabib et al., 2003), reversal learning in the BTBR (Molenhuis et al., 2014), and cued and contextual fear conditioning in BTBR (Stapley et al., 2013; MacPherson et al., 2008). The deficits found in the BTBR could be restored with an increase in training (Stapley et al., 2013) and cage enrichment (MacPherson et al., 2008). These results clearly indicate differences between the BTBR and B6 WT individuals in domains important in PKU research. It highlights that the fundaments on which the PKU genotype is induced are already different. Therefore, it is important to characterize the different strains' biochemical profile along with behavioral outcome in order to highlight similarities and, most of all, differences between the strains, providing better translational insight in the use of the PKU mouse model. For this reason, this study aims to directly compare male BTBR and B6 mice in terms of amino acid- and neurotransmitter-levels, and behavioral and cognitive performance under identical laboratory conditions (e.g., food regime, housing conditions, and experimental design). We particularly aimed to answer two specific questions: (1) Do WT and PKU mice of the two strains differ from each other? and (2) Do PKU mice differ from WT mice within a strain? To this aim, the mice were behaviorally tested in four PKU relevant domains previously described in the literature for at least one of the strains, namely (1) activity levels, (2) motor performance, (3) anxiety and/or depression-like behavior, and (4) learning and memory.

## Materials and methods

### Animals

**Figure 1:**
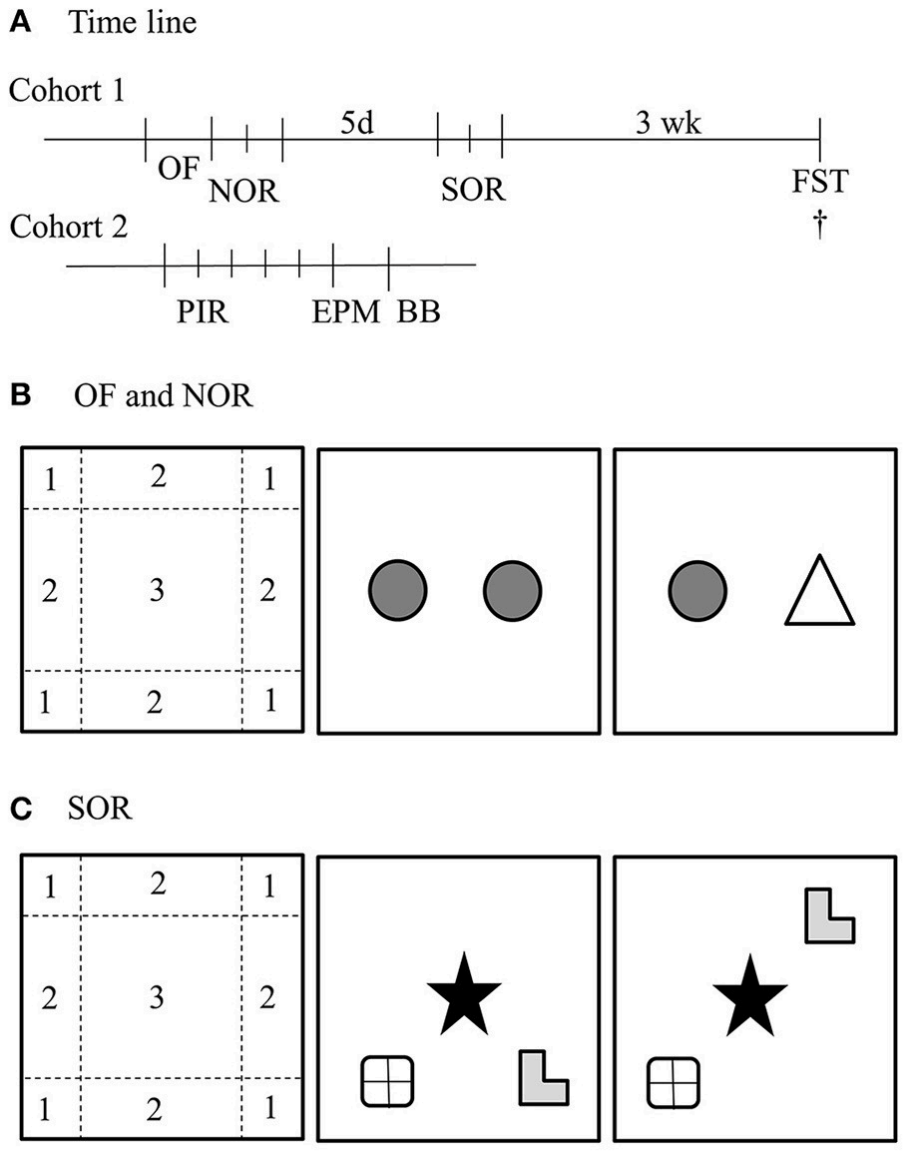
**Overview of behavioral paradigms. (A)** Time line of the study. The first cohort started with an open field (OF) and novel object recognition test (NOR), 5 days later followed by a spatial object recognition test (SOR) and 3 weeks later a Forced swim test (FST). After the FST, the mice were sacrificed. The second cohort was monitored for 5 days with passive infrared sensors (PIR), subsequently 24 h later tested in the elevated plus maze (EPM) and 24 h later on the balance beam (BB). **(B)** OF and NOR setup. The habituation phase of the NOR was used as OF. For the analysis with ethovision, the arena was divided in three regions; (1) corner, (2) border, and (3) center. The NOR was started 24 h after the habituation phase, in which the animals could freely explore two identical objects. Again 24 h later, one object was replaced for a new object. **(C)** The SOR was performed in 2 days. The first day the mice were exposed to four trials of 6 min with a 6 min break in between. The first trial was a habituation phase and the second to fourth trial the mice could explore three different objects in a specific configuration. 24 h later, one object was moved to another position. ^†^Depicts the time of euthanisation.

Heterozygous mating pairs of either BTBR or B6 mice were bred to obtain male BTBR WT, BTBR PKU, B6 WT, and B6 PKU mice. Original breeding pairs of B6 were obtained from the lab of Prof. Dr. Thöny, University of Zürich, Switzerland and in our hands crossed back every fifth generation with the C57Bl/6JRj (Janvier). The breeding pairs of the BTBR were kindly provided by Prof. Puglisi-Allegra, Sapienza University of Rome, Italy. They obtained the original BTBR-Pahenu2/J parental pairs from Jackson Laboratories (Bar Harbor, ME, USA) (Puglisi-Allegra et al., 2000) All individuals were weaned on postnatal day 28 and tissue obtained at weaning was used to establish genotype with quantitative PCR (forward primer: 5′ CCGTCCTGTTGCTGGCTTAC 3′, reverse primer: 3′ CAGGTGTGTACATGGGCTTAGATC 5, WT probe: CCGAGTCZZLCALTGCA, PKU probe: CCGAGTCZLLCACTGCA, aimed at exon 7 of the PAH gene (Eurogentec, Fremont, USA). Mice were group housed until the start of the experiment in a cage with a paper role and nesting material. Animals were tested around the age of 4–5 months. All mice were housed individually in cages with a paper role and nesting material 7 days before the experiment. Mice were handled by the researcher for 2 min on the 3 consecutive days before the start of the first behavioral paradigm. The reported behavioral paradigms were obtained from two separate cohorts of 10 males for each group. In the first cohort, in chronological order, we examined the open field (OF), long-term novel object recognition (NOR), long-term spatial object recognition (SOR), and the forced swim test (FST). In the second cohort, in chronological order, we examined home-cage activity, the elevated plus maze (EPM), and the balance beam (BB). An overview of the time line is given in Figure 1A. During the experiment, animals had *ad libitum* access to water and normal chow (RMH-B 2181, ABdiets, Phe: 8.7 g/kg food) and were kept on a 12/12 light/dark cycle. All experimental measurements, except for home-cage activity, were performed between Zeitgeber Time 1 (ZT1) and ZT6. All proceedings were carried out in accordance with the recommendation of the Guide for the Care and Use of Laboratory Animals of the National Institutes of Health (The ARRIVE Guidelines Checklist) and protocols were approved by the Institutional Animal Care and Use Committee of the University of Groningen (Permit No 6731A and 6731D).

### Amino acid and neurotransmitter analyses

Two hours after the FST, animals were anesthetized with isoflurane. When the hind paw reflex was no longer present, blood samples were taken via heart puncture and collected in heparin tubes (temporarily stored at 4°C). Brains were removed from the skull and cerebrum was immediately flash frozen. To obtain blood plasma, heparin tubes with the blood samples were centrifuged at 1500 rcf for 10 min and the supernatant was taken. Plasma and brain samples were stored at −80°C until further processing. Amino acid and neurotransmitter analyses were performed as described in van Vliet et al. (2015).

### Open field test

Activity and anxiety-like behavior were assessed in a square OF test (50 × 50 × 35 cm) with a white Plexiglas floor and gray Plexiglas walls with a checkerboard cue on one of the walls. This same arena was used for the NOR and SOR. In all tests with this arena, dim lighting was used (10 lux in the center of the arena). At the start of the trial, the animal was placed in the middle of the OF and left to explore the arena freely for 10 min. All trials were video recorded and were analyzed at a later time with Ethovision v.11. In this analysis, the arena was divided into a center zone, four border zones, and four corner zones (Hovens et al., 2014). Activity was quantified by the distance moved and anxiety-like behavior was examined by the preference of the animal to seek out the more sheltered zones.

### Novel object recognition

NOR was used to examine the ability of the mice to recall a familiar object after a delay of 24 h. This widely used learning and memory paradigm and the SOR task (see Section Spatial Object Recognition below) are both based on the innate exploratory behavior of mice toward novel stimuli. If mice successfully recall the previous event, they will increase exploration toward the novel stimuli and in the SOR a displaced object (Ennaceur and Delacour, 1988; Antunes and Biala, 2012). In Figure 1B is shown that the NOR test consists of three phases; the habituation phase, the familiarization phase, and the test phase (Antunes and Biala, 2012). The OF test was used as the habituation phase. The familiarization phase was performed 24 h after the habituation phase. Within this phase, the mice were allowed to freely explore two identical objects. Twenty four hours later, mice were exposed to one previously encountered object and one novel object in the test phase. The objects were randomized over the trials. All phases were 10 min long and video recorded. The read-out parameter of the NOR test was the time spent exploring the novel object compared to the previously encountered object, expressed as the ratio of novel object exploration time to total exploration time of both objects. The exploratory behavior was scored manually with the program ELINE (developed in house).

### Spatial object recognition

In the SOR test, mice were tested for their ability to recognize the displaced object after 24 h. Within this test, the displaced object that caused the new configuration of objects was the novel stimulus. SOR was tested 5 days after the NOR test. Comparable to the NOR test, the SOR test consisted of a habituation phase, a familiarization phase, and a test phase (Figure 1C). In the habituation phase, the mice could explore the arena without objects, similar to the previous OF. In the familiarization phases 2–4, the mice were placed in the same arena with three objects with different shapes and different materials (pottery, RVS, and glass) in a specific configuration. 24 h later, in the test phase, one of the outer objects was displaced (randomized between experimental groups). All trials were 6 min long and video recorded for analysis at a later time point. Between habituation phase and the familiarization phase, the mice were placed in their home cage and the arena was cleaned with 30% ethanol. All trials were manually scored for exploratory behavior with the program ELINE. The discrimination index for NOR was calculated by dividing the exploration time of the novel object by the total exploration time multiplied with 100. In contrast to NOR, in the SOR test it was not possible to keep all objects in the same distance from the walls and from each other in both familiarization and test phases, because the limitation of three objects in a rectangular space. Therefore, a correction was made for possible *a priori* preference for location or object. This correction was done by calculating the mean time spent on exploring each object in the three training sessions of the first day. These cumulative exploration times were set as a percentage of the overall exploration time. The difference between exploration time in the test trial and mean exploration time in the three training sessions was calculated and expressed as a percentage difference. A positive value depicts an increased preference of the individual toward the object in retrospect of possible innate preference at for hand.

### Forced swim test

The original FST (Porsolt et al., 1978) used to test antidepressants, consists of a pre-test session of 15 min and a test session of 5 min 24 h later. The now commonly used FST to assess depressive-like behavior without intervention consists of one trial (Bogdanova et al., 2013) and was used in this study. Mice were placed in a 5000 ml cylinder containing 2000 mL of 27°C tap water for 6 min. All trials were recorded with a video camera positioned in front of the cylinder. These videos were analyzed with ELINE and scored for the behaviors swimming, struggling, and floating. Swimming was defined as a controlled motion in which the animals used all four paws. Floating behavior was defined as an immobile state with only small movements of one paw to keep balance. Finally, struggling was defined as any other behavior to keep the head above water. This was often done with hasty movements of all four paws toward the side of the cylinder with a vertical body position. Literature shows that an increase in floating behavior is consistent with a more depression-like phenotype (Porsolt et al., 1978; Bogdanova et al., 2013).

### Home-cage activity

To examine spontaneous activity in a familiar environment, individuals were monitored with a passive infrared (PIR) detector in their home cage. After 7 days of individual housing, which included 3 days of handling, the animals were subjected to 5 consecutive days of PIR registration starting at ZT1. Starting at midnight, 4 days (96 h) were analyzed with ACTOVIEW (Mulder et al., 2013). This program calculates the average activity from the files produced by the activity management system (CAMS) collected from the PIR.

### Balance beam

The BB task was used to assess coordination and balance as previously described in Mazzola et al. (2016). In short, animals were trained over three distances (10, 40, and 75 cm) to cross a square wooden beam (length 1 m, width 5 mm, height 10 mm, horizontally positioned 50 cm above the underlying surface) to their home cage. This is followed by a read-out trial of 100 cm. At the start and between trials, the animals were left in their home cage for 1 min. In this study, the performance of the animal was measured by the number of correct steps as a percentage of the total steps necessary to cross the beam. A correct step was defined by the full placement of the hind paw on the beam from the initiation of the step to replacing it on the beam in a forward motion.

### Elevated plus maze

The second measurement of anxiety-like behavior is the EPM (Belzung and Griebel, 2001; Calabrese, 2008). This paradigm is also based on the innate exploratory behavior of the mice but this exploratory drive is counteracted by the reluctance of the mice to explore open and raised areas. The apparatus used for this paradigm was a plus-shaped maze with two open and two closed arms connected in the middle with an open center zone. The closed arms were surrounded by walls of 16 cm that were open at the top. The open arms solely had a small ridge (2 mm) surrounding the arms. The length of the arms was 29.5 cm and the center zone was 5 × 5 cm. The whole apparatus was positioned 50 cm above the ground. Low lighting conditions were used (30 lux open arms). At the start of the 8 min trial, the animals were placed with their head in the middle of the center zone, pointing toward an open arm. The trials were manually scored for the following parameters: number of open arm entries and closed arm entries, and the time spent in open arms and closed arms. The maze was divided into three zones: open arms, closed arms, and center. Entering into a new zone with four paws was seen as an entry and time recordings were taken from this point. A percentual score for each zone was calculated by dividing the time spent in a certain area by the total time.

### Statistical analysis

A one-way ANOVA was used in the statistical analysis of the data. When the one-way ANOVA resulted in a *p* ≤ 0.05, a Bonferroni *posthoc* test was performed. As the research question in the introduction did not focus on the comparison between PKU individuals and the opposing WT group, the statistical outcome of these comparisons (BTBR WT vs. B6 PKU and B6 WT vs. BTBR PKU) will not be discussed. To further investigate the learning and memory tasks, the discrimination index of each group was tested with a paired Student's *t*-test. A statistical significant difference was defined as *p* ≤ 0.05. Values two standard deviations outside the mean were viewed as outliers and discarded from the analysis.

## Results

### Phe measurements in blood and brain

**Figure 2:**
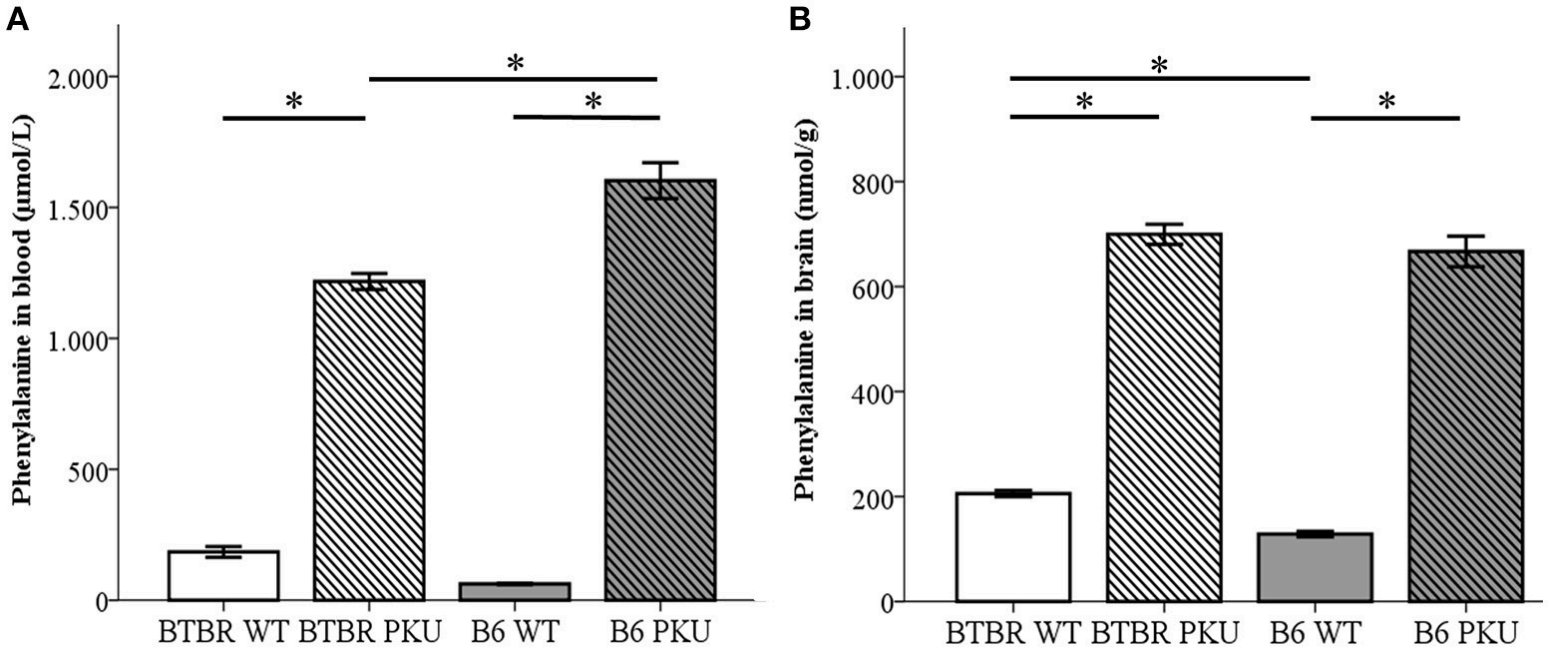
**Phenylalanine concentrations in blood and brain. (A)** Phenylalanine concentrations in blood (μmol/L) (*n* = 4–6), and **(B)** phenylalanine content in the brain (nmol/g) (*n* = 5–6) of BTBR and C57Bl/6 (B6) wild-type (WT) and phenylketonuria (PKU) mice. *p ≤ 0.05; mean ± SEM.

To examine if the identical point mutation in two different strains resulted in similar amino acid levels under the same food regimes, amino acid measurements were performed in blood and brain (Figures 2A,B). Phe levels differed between the groups in blood [*F*_(3, 17)_ = 106.129, *p* < 0.001] and brain [*F*_(3, 18)_ = 306.510, *p* < 0.001]. In blood, BTBR PKU showed Phe concentrations of 1270.8 ± 82.3 μmol/L, a 6.3-fold increase compared to the BTBR WT (Phe 200.8 ± 82.3 μmol/L, *p* < 0.001). In B6 PKU, a Phe concentration of 1632.5 ± 313.2 μmol/L was observed, which was a 26.2-fold rise compared to WT littermates (Phe 62.3 ± 7.7 μmol/L, *p* < 0.001). In respect to the blood Phe concentrations, brain Phe content in BTBR PKU mice were 699.5 ± 46.9 nmol/g wet weight and in B6 PKU 666.8 ± 65.4 nmol/g wet weight, resulting in, respectively, a 3.4-fold and 5.1-fold increase compared to WT littermates (BTBR WT 205.6 ± 13.3 nmol/g wet weight, B6 WT 128.5 ± 12.8 nmol/g wet weight, *p* < 0.001 for both). A comparison between strains showed that B6 PKU individuals had higher levels of Phe in blood than BTBR (*p* = 0.036), what was also found in the brain Phe levels between WT individuals (*p* = 0.034). Additional amino acids measurements in blood and brain are provided as Supplementary Material. In blood of the B6 strain, additional genotype differences were found in blood valine (*p* = 0.007), isoleucine (*p* = 0.001) and leucine (*p* = 0.002). In the BTBR strain, only differences were found in tyrosine (*p* < 0.001). The altered blood amino acid concentrations did not translate to similar changes in brain amino acid concentration. In brain, only tyrosine levels were reduced in B6 PKU compared to B6 WT (*p* = 0.001).

### Monoaminergic neurotransmitters in brain

**Figure 3:**
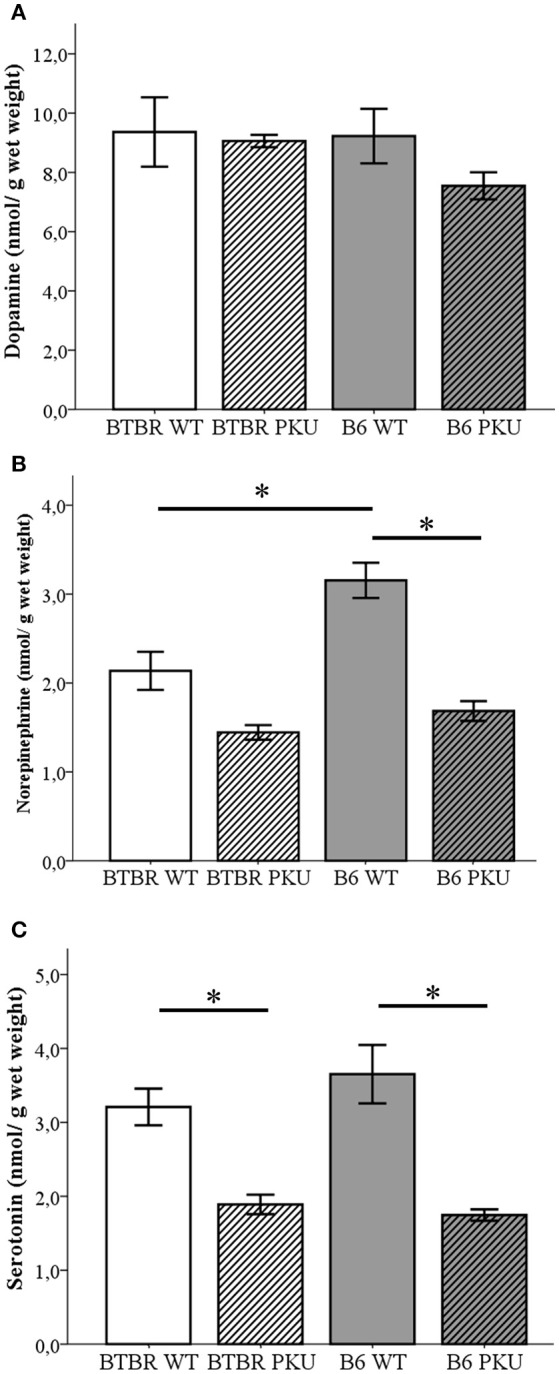
**Neurotransmitter analyses. (A)** Dopamine, **(B)** norepinephrine, **(C)** serotonin (*n* = 6 for all groups except for BTBR PKU *n* = 5). *p ≤ 0.05; mean ± SEM.

To further investigate the consequence on the biochemical level of the identical point mutation in both PKU strains, monoaminergic neurotransmitters together with the associated metabolite content were examined in the brain (Figures 3A–C). First, in Figure 3A, the catecholamine dopamine did not significantly differ between groups [*F*_(3, 19)_ = 1.094, *p* = 0.376]. Second, further downstream the catecholamine pathway, norepinephrine (NE) did show differences between the groups [Figure 3B; *F*_(3, 19)_ = 20.384, *p* < 0.001]. Norepinephrine levels were reduced to 53% in B6 PKU compared to WT littermates (*p* < 0.001) and a trend toward a reduction of 63% was observed in BTBR PKU (*p* = 0.061). B6 WT showed a higher norepinephrine content compared to BTBR WT (*p* = 0.002). Finally, in Figure 3C, serotonin levels showed differences between the groups [*F*_(3, 19)_ = 14.053, *p* < 0.001] in which BTBR PKU showed a 57% reduction (*p* = 0.012) and B6 PKU a 50% reduction (*p* < 0.001). No significant differences were observed between the WTs and PKUs of both strains (both: *p* = 1.000).

### Activity

**Figure 4:**
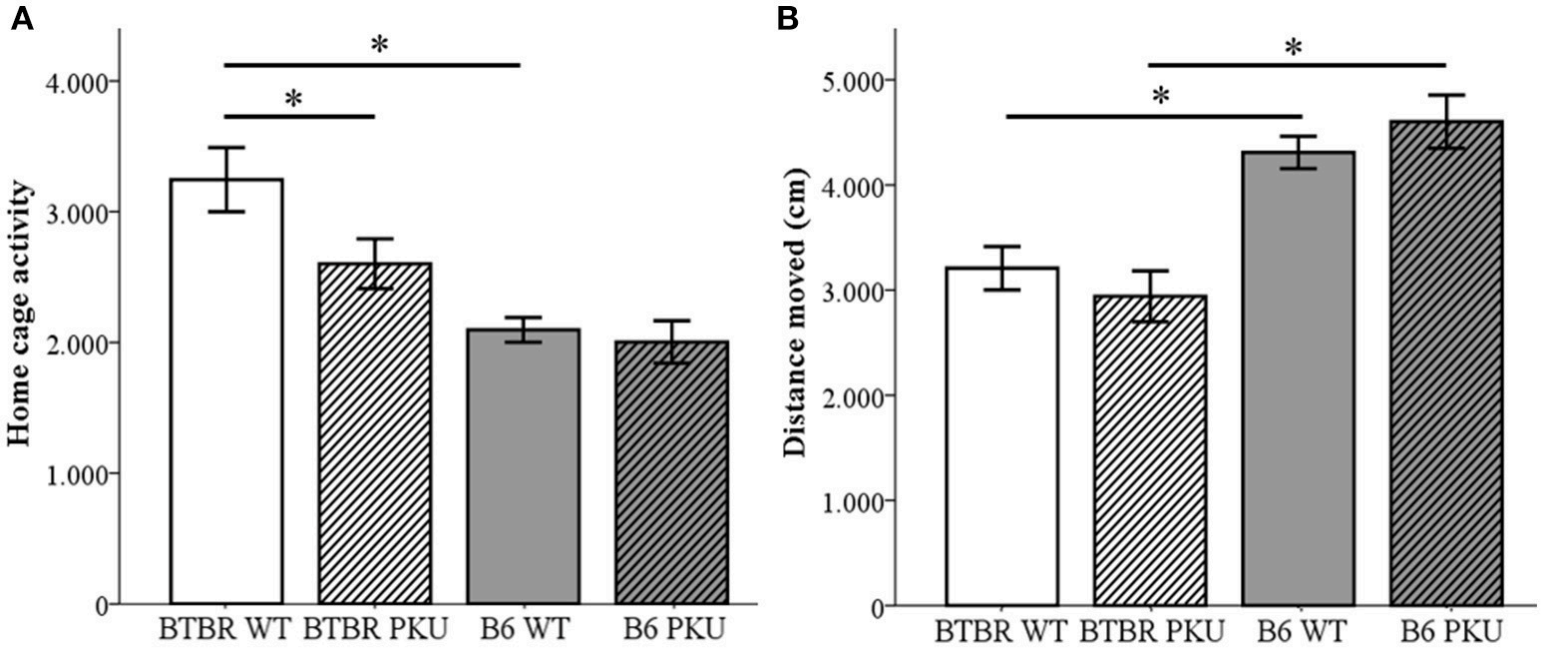
**Activity. (A)** Home-cage activity measured by PIR (*n* = 9–10). **(B)** Distance moved in an open field (*n* = 9–10). *p ≤ 0.05; mean ± SEM.

The behavioral phenotype was examined in four domains, starting with locomotor activity that was assessed in three experimental test conditions. First, home-cage activity measurements were taken to assess baseline locomotor activity (Figure 4A). A significant difference was observed between the WT of both strains [*F*_(3, 33)_ = 10.185, *p* < 0.001, BTBR WT vs. B6 WT, *p* < 0.001] and the PKU and WT individuals of the BTBR (*p* = 0.017). Such a difference was not observed in the B6 strain (*p* = 1.000). Second, the distance moved in an open field was measured to assess novelty-induced locomotion (Figure 4B). In this novel environment, a significant difference was found between strains [*F*_(3, 34)_ = 14.093, *p* < 0.001, BTBR WT vs. B6 WT *p* = 0.006, BTBR PKU vs. B6 PKU, *p* < 0.001] in which the B6 moved a greater distance. Furthermore, in contrast to the home-cage activity measurements, no significant differences were found in genotype (BTBR: *p* = 1.000, B6: *p* = 1.000). Finally, entries in the elevated plus maze were used to examine novelty-induced locomotion in a different arena. The results did not show significant differences between the groups [*F*_(3, 36)_ = 0.916, *p* = 0.443].

### Motor performance

**Figure 5:**
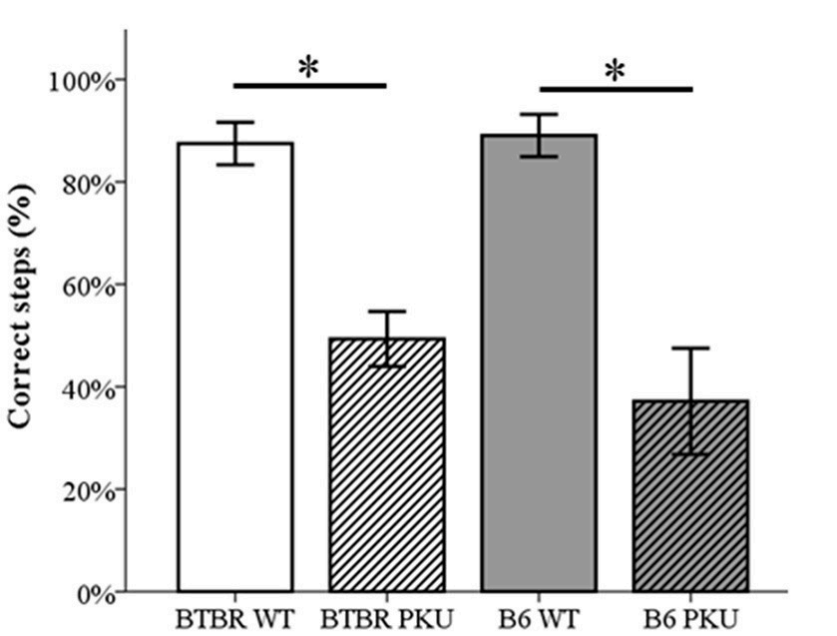
**Motor performance**. The number of correct steps in the probe trail is depicted as a percentage of the total steps necessary to cross the beam (*n* = 9–10). *p ≤ 0.05; mean ± SEM.

In Figure 5 it is clear that the motor performance, assessed by the percentage of correct steps in the read-out trial, differed significantly [*F*_(3, 36)_ = 16.479, *p* < 0.001, respectively]. In both strains, PKU individuals showed a lower percentage of correct steps compared to WT individuals (BTBR: *p* = 0.001, B6 *p* < 0.001). No significant differences were observed between strains (WTs *p* = 1.000, PKUs *p* = 1.000).

### Anxiety and depressive-like behavior

**Figure 6:**
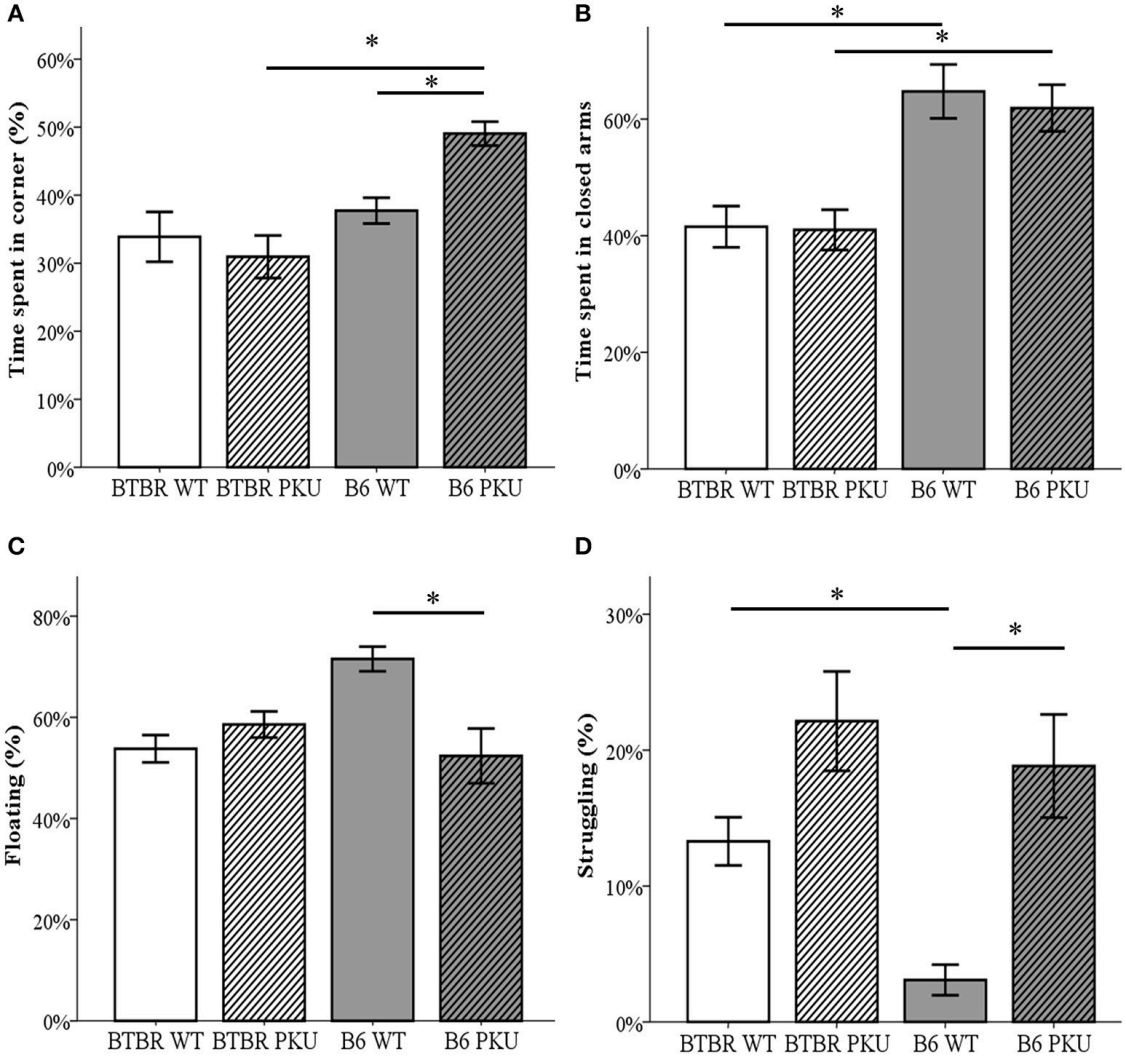
**Anxiety- and depressive-like behavior. (A)** The time spent in the corners of the open field, the most sheltered areas of the arena. The habituation phase of the NOR was used as open field (*n* = 9–10). **(B)** Time spent in the closed arms of the elevated plus maze (*n* = 10). **(C)** Floating behavior in the forced swim test (*n* = 9–10). **(D)** Struggling behavior in the forced swim test (*n* = 9–10). *p ≤ 0.05; mean ± SEM.

Anxiety and depressive-like behavior were examined by the use of the OF, the EPM, and the FST (Figures 6A–D). In the OF, time spent in the corners significantly differed between groups [*F*_(3, 34)_ = 9.164, *p* < 0.001]. B6 PKU significantly spent more time in the corners compared to the BTBR PKU (*p* < 0.001). Particularly, the B6 PKU individuals spent ~50% of the time in the corners which was more than their WT littermates (*p* = 0.024). In addition, the EPM showed a difference in the percentage of time spent in the closed arms [*F*_(3, 34)_ = 10.302, *p* < 0.001]. This significant difference was found between the strains (WTs *p* = 0.001, PKU's *p* = 0.004) but not between the PKU and WT individuals of each strain (BTBR: *p* = 1.000, B6: *p* = 1.000). Finally, the FST used to examine depressive-like behavior, showed significant differences in the amount of time spent on floating [*F*_(3, 34)_ = 6.155, *p* = 0.002), struggling [*F*_(3, 34)_ = 11.092; *p* < 0.001], and swimming [*F*_(3, 34)_ = 3.634, *p* = 0.022]. Floating and struggling differed only between B6 PKU and B6 WT but not between BTBR PKU and BTBR WT (floating: *p* = 0.003, *p* = 1.000, struggling; *p* = 0.002, *p* = 0.140, respectively). In swimming, only a significant reduction of swimming behavior was observed in BTBR PKU individuals compared to BTBR WT (BTBR: *p* = 0.047, B6 *p* = 0.221).

### Learning and memory

**Figure 7:**
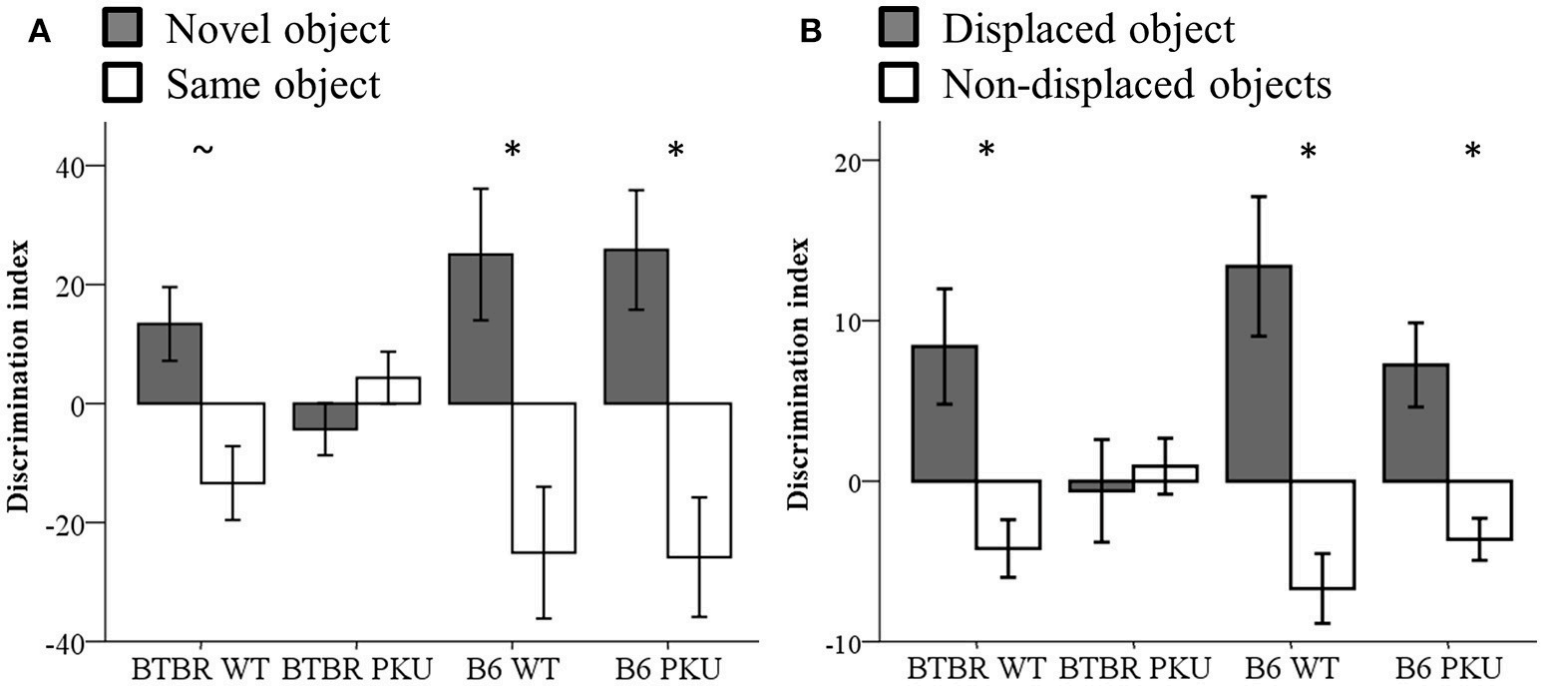
**Learning and memory. (A)** Discrimination index of NOR (*n* = 10). **(B)** Discrimination index of SOR (*n* = 8–10). *p ≤ 0.05, ~*p* = 0.06; mean ± SEM.

Learning and memory were assessed by the NOR and SOR tests (Figures 7A,B). The performance on the discrimination index of the novel object and the displaced object showed a trend between groups [NOR: *F*_(3, 36)_ = 2.818, *p* = 0.053], SOR: [*F*_(3, 34)_ = 2.775, *p* = 0.056]. To test whether each group learned the task, a paired *t*-test was used to examine if either the novel object differed from the same object or the displaced object from the non-displaced object. In NOR, the B6 WT [*t*_(9)_ = −2.265, *p* = 0.050] and B6 PKU [*t*_(8)_ = 2.762, *p* = 0.030] learned the task. Within the BTBR WT a trend was observed [*t*_(9)_ = −2.153, *p* = 0.060]. The PKU BTBR did not learn the task [*t*_(9)_ = 0.986, df = 9, *p* = 0.350]. In SOR, BTBR WT [*t*_(9)_ = 2.335, *p* = 0.044], B6 WT [*t*_(8)_ = 3.076, *p* = 0.015], and B6 PKU [*t*_(8)_ = 2.762, *p* = 0.025] learned the task. Again, PKU BTBR mice did not learn the task (*t* = −0.314, df = 9, *p* = 0.761). No significant differences were found in the exploration of the objects in the NOR test [*F*_(3, 36)_ = 1.093, *p* = 0.364]. In the test session of the SOR, the BTBR mice explored the objects overall more than the B6 mice [*F*_(3, 36)_ = 5.463, *p* = 0.003, BTBR compared to B6 *p* < 0.001].

## Discussion

Here, we directly compared the two strains of the PKU mouse model in behavioral domains previously described for at least one of the two strains in literature (activity levels, motor performance, anxiety and/or depression-like behavior, and learning and memory) and PKU-related biochemical parameters in the same laboratory using the same experimental settings. Distinct differences in behavioral outcome between the two strains were found in all four above-mentioned domains, regardless of comparable biochemical changes in amino acid and neurotransmitter content. The result of the mutation in the PAH enzyme on behavior was most pronounced in the PKU BTBR mice, revealing changes in home-cage activity, reduced motor performance, and learning and memory deficits. In contrast, compared to B6 WT mice, PKU B6 mice only showed reduced motor performance and indications of differences in anxiety-like behavior. Therefore, differences in the phenotypical outcome of the BTBR and B6 PKU mouse model seem to be primarily due to factors inherent to the genetic background of the mouse and much less to differences in biochemical parameters in blood and brain that are typically described for PKU pathology.

### Learning and memory are intact in B6 PKU

On a behavioral level, the cognitive outcome is consistently used to assess the severity of the PKU pathology and effectiveness of (new) treatments. This study confirms the cognitive deficits described for BTBR PKU mice in the literature. However, we show for the first time that B6 PKU mice can master a learning and memory paradigm despite severe disruptions in amino acid and neurotransmitter content in the brain. The differences in phenotypical behavior could lay in a different ability to understand the cognitive demands of a learning and memory paradigm of the genetic background. In the literature, a direct comparison between BTBR WT and B6 WT shows mixed results. As mentioned in the introduction, some articles show an intact ability to master a short-term or long-term memory task by both strains (Cabib et al., 2003; Molenhuis et al., 2014). In contrast, memory deficits in short-term novel object memory in the B6 (Cabib et al., 2003), reversal learning in the BTBR (Molenhuis et al., 2014), and cued and contextual fear conditioning in BTBR (MacPherson et al., 2008; Stapley et al., 2013) have been reported. The deficits found in BTBR could be restored with an increase in training (Stapley et al., 2013) and cage enrichment (MacPherson et al., 2008). In our study, difficulties to master the learning and memory paradigms by BTBR WT were also found for the NOR but not for the SOR. This outcome could have been influenced by the order of testing (the NOR always preceded the SOR), or the number of training sessions (a single training phase in NOR, vs. three training phases in SOR). Our results together with literature suggest that BTBR WT mice have more difficulties mastering a learning and memory paradigm compared to B6 WT mice. It is up for discussion whether and to what degree the PKU behavioral phenotype in the BTBR PKU is a result of weakening a poor learner, creating a deficit, or that the strong learner (B6 WT) is able to compensate.

### Can PKU-related biochemical changes affect both strains differently?

Both strains showed a vast increase in Phe levels and disrupted serotonin and norepinephrine levels in the brain (for norepinephrine a trend was observed for BTBR statistically) which is in accordance with previous studies (Andolina et al., 2011; Pascucci et al., 2013; Sawin et al., 2014; van Vliet et al., 2015). How these PKU-related changes can result in a different functional outcome is not clear. Concerning raised Phe concentrations, in *in vitro* models of PKU, increased Phe concentrations seems to affect post- and presynaptic markers, proteins involved in cytoskeleton organization, and neuronal morphology (Zhang and Gu, 2005; Hörster et al., 2006; Zhang et al., 2007; Li et al., 2010; Horling et al., 2015; Schlegel et al., 2016). *In vivo*, although both strains show changes in different markers related to synaptic functioning, both BTBR PKU and B6 PKU show affected synaptic plasticity and overall neuronal functioning (Andolina et al., 2011; Liang et al., 2011; Horling et al., 2015; Bruinenberg et al., 2016). Differences are found between BTBR WT and B6 WT in adult neurogenesis and neurodevelopmental markers but not in the synaptic markers synaptophysin and postsynaptic density protein (PSD-95) that are discussed in PKU literature (Stephenson et al., 2011). The reduced neurogenesis together with altered neurodevelopmental markers in BTBR WT indicates that BTBR could have a different brain development compared to B6 WT (Stephenson et al., 2011). As changes in Phe and neurotransmitters are chronically present at a very early age, we assume that neurodevelopment differs between BTBR PKU and B6 PKU. An indication that this is the case is given by the work of Andolina et al. (2011). In their study, they could improve dendritic spine maturation and performance in a short-term version of the NOR and SOR tests with a 7-day treatment (PND-14-21) with 5-hydroxytryptophan, a precursor of serotonin (Andolina et al., 2011). It is not clear if B6 mice only have a different neonatal development or that they also have different susceptibility toward neurotransmitter depletion in life. Some indications present in literature suggest that B6 can differ from BTBR in their response to neurotransmitter manipulations. For instance, a comparison of acute tryptophan depletion, a method to deplete serotonin, both B6 and BTBR WT's show a decrease in serotonin but the tryptophan depletion only altered social interaction and social novelty behaviors in B6 and not in BTBR (Zhang et al., 2015). Furthermore, administration of the serotonin agonist 3-chlorophenylpiperazine (CCP) in WT B6 has not affected locomotor activity nor performance of the B6 in learning and memory paradigm (Vetulani et al., 1982). Finally, in a conditional knock-out mouse maintained on a B6 genetic background, a significant decrease in serotonin and norepinephrine concentrations did not affect locomotor activity, motor performance, and anxiety- and depression-related behaviors (Isingrini et al., 2016). These examples highlight that in addition to the differences found between BTBR WT and B6 WT in neurodevelopment, the consequence of neurotransmitter depletion in later life (without differences in development) could be different between BTBR and B6. Therefore, we hypothesize that the PKU-related changes in Phe and neurotransmitters affect both strains differently during neurodevelopment and later in life resulting in a difference in phenotypical behavior.

### Considerations in testing behavior

In this study, we identified differences between PKU of the B6 background and the PKU of the BTBR background. As all domains seem to be affected to some extent, an important consideration is the possibility that altered behavior in one domain can influence the outcome nonspecifically in another behavioral paradigm. For example, deficits found in motor performance in the BB of both genetic backgrounds could influence the outcome in the OF, EPM, SOR, NOR, and FST. However, we did not clearly find an influence of motor performance in behavioral paradigms with a low level of required motor performance (OF, EPM, NOR, SOR) as both strains did not reduce activity in the OF and the EPM, and no differences were found in exploration of the objects in the NOR. Therefore, the mice were not hampered to fulfill the key feature of the task. However, the task that required most motor skills, i.e., the FST, could be affected by the motor problems found in PKU. Accordingly, we observed that the PKU mice from both backgrounds showed difficulties in maintaining a floating position and correct swimming behavior during the FST. As a result, we believe that the changes found in the FST are primarily attributed to deficits in motor performance. Therefore, we conclude that the FST in PKU mice is not well suited for examining depressive-like behavior in PKU mice and conclusions in this domain should, therefore, be drawn with caution.

### The translational value of the PKU mouse model

The differences in behavioral outcome between PKU mice of both strains emphasizes that the consequences of the PAH mutation are influenced by other factors than Phe levels alone. Although the underlying mechanisms may be different, B6 PKU mice may resemble the human situation where some specific untreated PKU patients with high blood Phe concentrations have clearly escaped from the severe symptoms of PKU (Ramus et al., 1993).

To conclude, this study showed clear differences in PKU behavioral phenotype between BTBR and B6 mice despite similar biochemical phenotype. It contributes to a better translational insight in the use of the PKU mouse model. Future research should consider these differences when choosing one of the genetic strains to investigate the underlying mechanisms of PKU and/or new treatment targets. As the origin of BTBR and B6 PKU strains between labs may differ (or the frequency of backcrossing), our results also stress the need for a better genetic understanding of these strains used by research groups worldwide. Nevertheless, we would like to emphasize that both PKU strains have their own translational value for studying PKU and developing novel interventional strategies to battle the burden of the disease, as they may represent different patient populations.

## Author contributions

The study was designed by VMB, DvV, MJdG, FJvS, and EAvdZ. The breeding and the behavioral testing were performed by MJdG, VMB, and PNM. The analysis of behavioral studies and tissue together with the interpretation was done by DvV, EvdG, PNM, MJdG, VMB, MvF, and MRH-F. VMB drafted the manuscript that was critically revised by EAvdZ, FJvS, DvV, EvdG, MJdG, and PNM. All authors approved the final version of this manuscript.

### Conflict of interest statement

The authors declare that the research was conducted in the absence of any commercial or financial relationships that could be construed as a potential conflict of interest.
