# Supplementary material for: The Behavioral Consequence of Phenylketonuria in Mice Depends on the Genetic Background
Source: Front Behav Neurosci. 2016 Dec 20;10:233. doi: 10.3389/fnbeh.2016.00233 (PMC5167755; doi:10.3389/fnbeh.2016.00233)
Supplement: Supplementary file 1 [file Table1.DOCX]

Supplementary Material

The Behavioral Consequence of Phenylketonuria in Mice Depends on the Genetic Background

Vibeke M. Bruinenberg, Els van der Goot, Danique van Vliet, Martijn J. de Groot, Priscila N. Mazzola, M. Rebecca Heiner-Fokkema, Martijn H. van Faassen, Francjan J. van Spronsen, Eddy A. van der Zee*.

*** Correspondence:** Eddy A. van der Zee: e.a.van.der.zee@rug.nl

## Supplementary Tables

**Table 1. Amino acid concentrations in blood (µmol/L).** Each column depicts the mean concentration of the amino acids ± standard error of the mean. The Greek symbols are used to highlight significant differences; α= a difference between BTBR WT and BTBR PKU, β= a difference between B6 WT and B6 PKU, γ= a difference between BTBR PKU and B6 PKU (n=4-6).

|  | BTBR |  |  |  |  |  |  | B6 |  |  |  |  |  |  |  |
| --- | --- | --- | --- | --- | --- | --- | --- | --- | --- | --- | --- | --- | --- | --- | --- |
|  | WT |  |  | PKU |  |  |  | WT |  |  |  | PKU |  |  |  |
| Phenylalanine | 200.8 | ± | 36.8 | 1270.8 | ± | 43.8 | ^α^ | 58.3 | ± | 1.0 |  | 1632.5 | ± | 127.9 | ^β,γ^ |
| Tyrosine | 65.4 | ± | 2.8 | 27.8 | ± | 5.5 | ^α^ | 53.0 | ± | 1.7 |  | 38.0 | ± | 8.1 |  |
| Valine | 247.8 | ± | 11.6 | 237.0 | ± | 15.1 |  | 181.2 | ± | 4.2 |  | 277.2 | ± | 28.7 | ^β^ |
| Isoleucine | 89.8 | ± | 3.0 | 92.5 | ± | 6.7 |  | 68.7 | ± | 4.7 |  | 104.3 | ± | 6.3 | ^β^ |
| Leucine | 148.6 | ± | 6.6 | 140.3 | ± | 10.9 |  | 117.8 | ± | 4.9 |  | 170.0 | ± | 11.2 | ^β^ |
| Histidine | 62.8 | ± | 3.1 | 65.0 | ± | 1.8 |  | 51.2 | ± | 1.0 |  | 67.7 | ± | 7.9 |  |
| Threonine | 144.2 | ± | 9.6 | 131.5 | ± | 3.7 |  | 116.7 | ± | 2.9 |  | 156.5 | ± | 19.2 |  |

**Table 2. Amino acid concentrations in brain (**nmol/g**).** Each column depicts the mean concentration of the amino acids ± standard error of the mean. The Greek symbols are used to highlight significant differences; α= a difference between BTBR WT and BTBR PKU, β= a difference between B6 WT and B6 PKU, γ= a difference between BTBR PKU and B6 PKU, δ= a difference between BTBR WT and B6 WT (n=5-6).

|  | BTBR |  |  |  |  |  |  | B6 |  |  |  |  |  |  |  |
| --- | --- | --- | --- | --- | --- | --- | --- | --- | --- | --- | --- | --- | --- | --- | --- |
|  | WT |  |  | PKU |  |  |  | WT |  |  |  | PKU |  |  |  |
| Phenylalanine | 205.6 | ± | 5.4 | 699.5 | ± | 21.0 | ^α^ | 128.5 | ± | 5.7 | ^δ^ | 666.8 | ± | 26.7 | ^β^ |
| Tyrosine | 125.4 | ± | 3.8 | 107.8 | ± | 8.8 |  | 128.0 | ± | 8.9 |  | 81.6 | ± | 0.6 | ^β^ |
| Valine | 84.4 | ± | 5.8 | 94.5 | ± | 9.6 |  | 93.2 | ± | 3.9 |  | 67.4 | ± | 4.3 | ^γ^ |
| Isoleucine | 53.0 | ± | 3.1 | 65.0 | ± | 7.9 |  | 54.0 | ± | 3.8 |  | 48.6 | ± | 2.1 |  |
| Leucine | 198.4 | ± | 7.8 | 224.2 | ± | 18.6 |  | 200.7 | ± | 9.2 |  | 175.8 | ± | 3.3 |  |
| Histidine | 90.4 | ± | 4.0 | 124.0 | ± | 4.6 |  | 86.7 | ± | 4.9 |  | 95.2 | ± | 2.2 | ^γ^ |
| Threonine | 370.2 | ± | 7.7 | 347.8 | ± | 14.4 |  | 347.3 | ± | 17.1 |  | 300.2 | ± | 13.1 |  |
| Methionine | 80.0 | ± | 7.4 | 93.8 | ± | 10.2 |  | 77.3 | ± | 4.5 |  | 62.8 | ± | 3.3 | ^γ^ |
| Tryptophan | 10.2 | ± | 1.6 | 12.3 | ± | 2.0 |  | 13.0 | ± | 1.8 |  | 10.2 | ± | 2.2 |  |

**
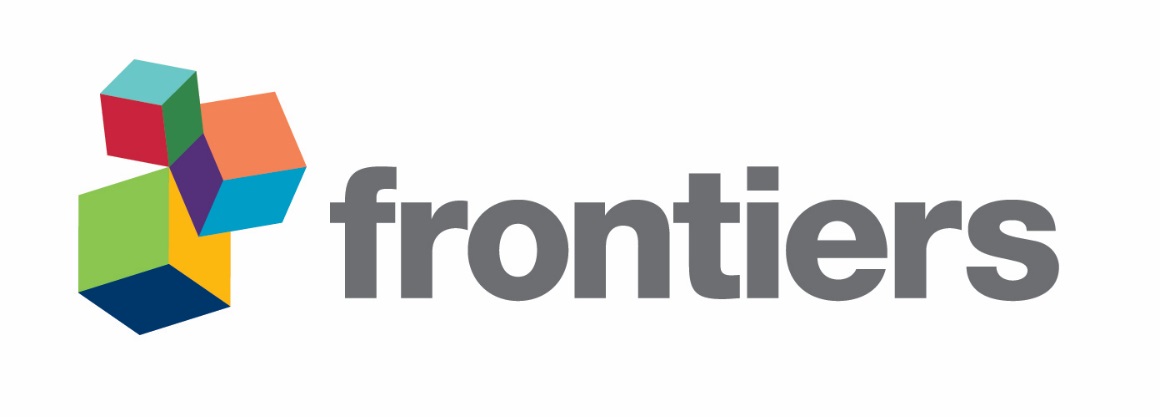
**
